# Adaptation improves face trustworthiness discrimination

**DOI:** 10.3389/fpsyg.2013.00358

**Published:** 2013-06-19

**Authors:** B. D. Keefe, M. Dzhelyova, D. I. Perrett, N. E. Barraclough

**Affiliations:** ^1^Department of Psychology, University of YorkYork, UK; ^2^School of Psychology, University of St AndrewsSt Andrews, UK

**Keywords:** face adaptation, face trustworthiness, face discrimination, adaptation, psychological, face perception, functional benefit

## Abstract

Adaptation to facial characteristics, such as gender and viewpoint, has been shown to both bias our perception of faces and improve facial discrimination. In this study, we examined whether adapting to two levels of face trustworthiness improved sensitivity around the adapted level. Facial trustworthiness was manipulated by morphing between trustworthy and untrustworthy prototypes, each generated by morphing eight trustworthy and eight untrustworthy faces, respectively. In the first experiment, just-noticeable differences (JNDs) were calculated for an untrustworthy face after participants adapted to an untrustworthy face, a trustworthy face, or did not adapt. In the second experiment, the three conditions were identical, except that JNDs were calculated for a trustworthy face. In the third experiment we examined whether adapting to an untrustworthy male face improved discrimination to an untrustworthy female face. In all experiments, participants completed a two-interval forced-choice (2-IFC) adaptive staircase procedure, in which they judged which face was more untrustworthy. JNDs were derived from a psychometric function fitted to the data. Adaptation improved sensitivity to faces conveying the same level of trustworthiness when compared to no adaptation. When adapting to and discriminating around a different level of face trustworthiness there was no improvement in sensitivity and JNDs were equivalent to those in the no adaptation condition. The improvement in sensitivity was found to occur even when adapting to a face with different gender and identity. These results suggest that adaptation to facial trustworthiness can selectively enhance mechanisms underlying the coding of facial trustworthiness to improve perceptual sensitivity. These findings have implications for the role of our visual experience in the decisions we make about the trustworthiness of other individuals.

## Introduction

Prolonged exposure to a visual stimulus can alter the tuning of neurons that encode that stimulus by a process known as adaption (Barlow and Hill, 1963). A consequence of this process is that the perception of subsequently viewed visual stimuli is biased in the opposite direction to the adaptor. For example, after adapting to a leftward moving grating, subsequently viewed gratings can appear to move in a rightward direction. These perceptual biases, known as aftereffects, have been demonstrated following adaptation to stimuli as diverse as orientation (Gibson and Radner, 1937), speed (Goldstein, 1957), contrast (Ross et al., 1993), spatial frequency (Blakemore and Campbell, 1969), facial configuration (Webster and MacLin, 1999), biological motion (Jordan et al., 2006; Troje et al., 2006), actions (Barraclough et al., 2009), and complex natural scenes (Greene and Oliva, 2010).

Although such biases in perception appear to be maladaptive, adaptation can calibrate the system to the population of stimuli to which it is exposed, making efficient use of a limited neural bandwidth. For example, adapting to a stimulus of constant velocity distorts the speed at which the stimulus is perceived, but increases sensitivity to changes in velocity (Clifford and Langley, 1996). Thus, adaptation allows for increased differential sensitivity at the cost of absolute sensitivity. Being able to detect smaller differences around the adapted level (average input) is clearly advantageous and shows the functional benefit of adaptation. Furthermore, this differential sensitivity increases as a function of adaptation duration, allowing us to detect even smaller differences to stimuli to which we are commonly exposed (Clifford and Langley, 1996).

Improved discrimination following adaptation has been demonstrated for relatively simple stimuli, coded by lower-level visual processing mechanisms, such as motion (Phinney et al., 1997), speed (Clifford and Langley, 1996), and orientation (Clifford et al., 2001). Adaptation to more complex stimuli, like faces, is thought to result from adaptation acting on mechanisms at a high-level in the visual system where faces are represented. Face aftereffects, however, show many similar characteristics to lower-level aftereffects, including a logarithmic build up with exposure to the adapting stimulus and a logarithmic decay over time (Leopold et al., 2005; Rhodes et al., 2007a). Recently a number of studies have examined whether face adaptation can also enhance sensitivity for faces, however, results have been equivocal. Rhodes et al. (2007b) found no improvement in sensitivity to facial identity following adaption to an average face (but see Wilson et al., 2002). Similarly, studies into adaptation on facial gender and ethnicity also failed to find any improvement in sensitivity (Ng et al., 2008). More recently, adaptation to both facial gender (Yang et al., 2011) and face viewpoint (Chen et al., 2010) have been shown to improve sensitivity around the adapted level. In addition, Rhodes et al. (2010) have demonstrated that face adaptation can lower identification thresholds to an adapted race (Asian or Caucasian), a finding that offers insight into the own-race bias.

In this study we tested if adapting to facial trustworthiness can improve trustworthiness discrimination. Trustworthiness is a multi-dimensional judgment and correlates highly with the valence of the face, with happy faces being perceived as trustworthy and angry faces as untrustworthy (Todorov et al., 2008; Sutherland et al., 2013). Adapting to angry or happy faces results in neutral faces being judged as more trustworthy or untrustworthy, respectively (Engell et al., 2010), demonstrating a role of emotion adaptation on facial trustworthiness. Furthermore, adapting to facial trustworthiness has a direct influence on the subsequent perception of facial trustworthiness (Wincenciak et al., 2013). Wincenciak et al. showed that exposure to trustworthy and untrustworthy faces resulted in repulsive aftereffects in female observers, where subsequent test stimuli appeared less like the adapting stimuli. In contrast, trustworthiness adaptation appeared not to bias face perception in male observers. Although this shows the capacity for trustworthiness adaptation to bias perception in female observers, we wanted to examine the potential benefit of improved trustworthiness discrimination following adaptation in both female and male observers.

We examined whether adapting to different levels of facial trustworthiness increases sensitivity around the adapted level. Three experiments were performed. In the first experiment we measured trustworthiness discrimination thresholds for an untrustworthy female face after participants adapted to an untrustworthy female face, a trustworthy female face, or did not adapt. In the second experiment we measured trustworthiness discrimination thresholds to a trustworthy female face, using the same adaptation conditions as in experiment 1. In the third experiment we examined whether adapting to an untrustworthy male face would improve discrimination to an untrustworthy female face. The third experiment was conducted to examine if any improvement in sensitivity transfers across changes in gender and identity as would be expected if an identity-independent representation of trustworthiness is being adapted.

## Methods

### Participants

Participants were University of York students and staff. All had normal or corrected to normal vision. Participants gave informed consent and were paid for their participation. Experiments were approved by the ethics committee of the Department of Psychology, University of York, and were performed in accordance with the ethical standards laid down in the 1990 Declaration of Helsinki.

Twelve participants took part in experiment 1 (6 female, mean age = 27, *SD* = 3.6). Ten of the participants from experiment 1 took part in experiment 2 (5 female, mean age = 28, *SD* = 3.24). Fifteen participants took part in experiment 3 (6 female, mean age = 29, *SD* = 7.9), 7 of whom had taken part in experiment 1, and 6 of whom had taken part in experiment 2. All participants were naive to the aims of the study, except in experiments 1 and 2 where one of the authors was a participant (B. D. Keefe), and in experiment 3, where two of authors were participants (B. D. Keefe and N. E. Barraclough).

### Stimuli

**Figure 1:**
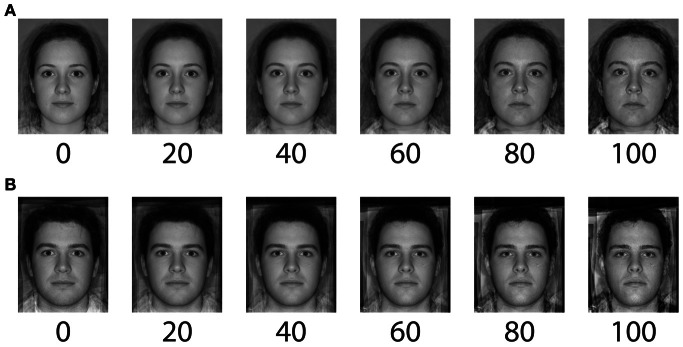
**The continua of (un)trustworthy face stimuli shown as a percentage of the morph level.** Stimuli were generated by morphing between the trustworthy prototype (illustrated here as the 0 face) and the caricatured untrustworthy prototype (illustrated here as the 100 face). Female faces **(A)**, and male faces **(B)**.

Face stimuli were obtained from The Perception Lab, University of St Andrews. The original set of stimuli included 99 faces (49 male) of Caucasian students, age range 17 to 25, displayed on a white background with a neutral expression, minimal makeup and no jewelry, and were horizontally aligned and scaled to the same interpupillary distance. Each face was rated for trustworthiness using a 7-point Likert scale by independent observers. Untrustworthy and trustworthy face prototypes generated by averaging (Rowland and Perrett, 1995) separately the 8 most untrustworthy and the 8 most trustworthy faces of each sex from the bank of 99 images. To generate female and male faces that varied on the level of trustworthiness that they conveyed, we morphed between each of the two same sex prototypes (Tiddeman et al., 2001). First, for both female and male faces we created caricatures of the untrustworthy face by generating new faces conveying 50% more untrustworthiness than the untrustworthy prototypes. Second, for each gender, we generated a continuum of 101 faces by morphing between the trustworthy prototype and the untrustworthy caricature. Each face stimulus on this continuum (see Figure 1) conveys a particular level of untrustworthiness, and this is expressed as the percentage level of the morph.

### Experimental procedures

A PC running Matlab 2010a (The MathWorks, Natick, MA) and Psychtoolbox (Brainard, 1997; Pelli, 1997; Kleiner et al., 2007) was used to control the experiment, display the stimuli, and record participants responses. Participants sat in a dimly lit room ~57 cm away from a 24 in TFT monitor (Acer GD245HQ, 1920 × 1080 pixels, 100 Hz refresh rate) on which all visual stimuli were presented. We measured trustworthiness-discrimination thresholds (JNDs) using a 2-IFC procedure.

**Figure 2:**
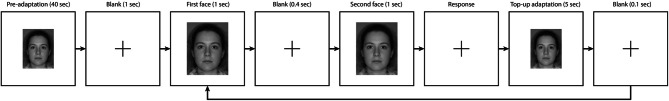
**Overview of the experimental procedure**.

In experiment 1 JNDs were measured for an untrustworthy female face (80) under 3 conditions: after adapting to an untrustworthy female face (80), after adapting to a trustworthy female face (40), or without adaptation. The adaptation procedure is illustrated in Figure 2. An initial 40 s of pre-adaptation was followed by a 1 s blank interval. Following the interval two test faces (a standard and a comparison face) were presented for 1 s each, with a 400 ms inter-stimulus interval. The screen then went blank and participants indicated which of the two faces was more untrustworthy using a key press. On all following trials the test faces were preceded by 5 s of top-up adaptation, followed by a blank screen for 100 ms. For the no adaptation condition, participants completed the same 2-IFC procedure without any adaptation. A fixation cross was displayed at the center of the monitor during blank intervals, and participants were required to maintain fixation. The degree of untrustworthiness conveyed by the standard face was always 80 and the degree of untrustworthiness conveyed by the comparison face was varied using adaptive staircase procedures. Participants completed each condition with each of 2 interleaved staircase reversal rules (1-up, 2-down; 2-up, 1-down). We did not determined thresholds from the staircase endpoints; these procedures were used to distribute trials at informative points along the psychometric function, which was fitted using the data from all trials. The step size was initially 8%, and was halved on each of the first 3 reversals. The staircase quit after 14 reversals, typically resulting in ~45 trials per staircase type (~90 trials per psychometric function). The order of the standard and comparison within each trial was randomized. Participants adapted to each condition in separate testing blocks with at least 5 min between blocks. The order of testing block was counterbalanced across participants. To avoid local (feature) adaptation, the adapting stimulus was 75% the size of the test stimulus (adapting stimulus subtended ~7.4 × 9.4°; test stimulus subtended ~9.9 × 12.6°).

In experiment 2, we measured JNDs for a trustworthy female face (40) after participants adapted to a trustworthy female face (40), adapted to an untrustworthy female face (80), or without adaptation. We chose to use the trustworthy female face (40) rather than an even more trustworthy female face (e.g., 20) to ensure that participants were able to perform the discrimination task. People are better at discriminating untrustworthy faces (Oosterhof and Todorov, 2008) therefore by using the trustworthy female face (40) as the standard face, a greater range of comparison trustworthy female faces were available during the adaptive staircase. Otherwise, the experimental procedure was identical to that used in experiment 1.

In experiments 1 and 2 the adapting and test faces were similar on multiple dimensions other than trustworthiness (e.g., identity and gender). Conceivably identity adaptation (Leopold et al., 2001; Rhodes and Jeffery, 2006; Rhodes et al., 2010) could explain any effect of adaptation to our untrustworthy stimuli. In order to rule out this possibility we conducted a third control experiment (Experiment 3) where we examined whether adapting to an untrustworthy male face would improve discrimination of an untrustworthy female face (80).

**Figure 3:**
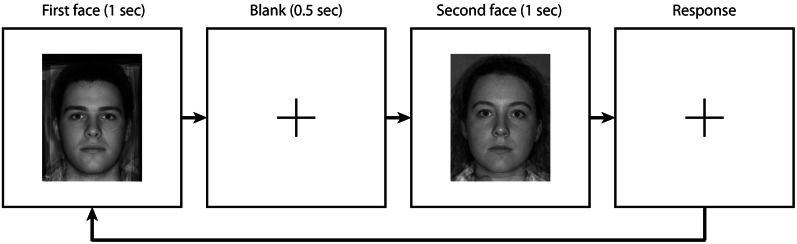
**Overview of the experimental procedure used to derive the PSE of the male face in experiment 3**.

To account for individual difference in the perception of trustworthiness conveyed by male and female faces, for each participant we matched perceived trustworthiness between the male adaptor face and the female standard test face. Each participant first completed a 2-IFC procedure to measure their point of subjective equality (PSE) between the untrustworthy female face (80) and male faces. A method of constant stimuli was used in which the standard was always an untrustworthy female face (80; see Figure 1A). The comparison was always a male face from the male trustworthiness continuum (see Figure 1B). Nine male comparison faces ranging from 60 to 100 in 5% steps were used. On each trial the two test faces were presented for 1 s each, separated by a 500 ms inter-stimulus interval. The screen then went blank and participants indicated which of the two faces was more untrustworthy using a key press (Figure 3). The order of the standard and the comparison was randomized on each trial.

For each participant the psychometric function was fitted to data from 90 trials (10 for each level of the comparison). The PSE was defined as the 50% point of the psychometric function and represents the point at which the male face was perceived with the same level of trustworthiness as the standard female face (80). Each participant's PSE was subsequently used to determine the degree of untrustworthiness conveyed by the male adapting face for each participant.

During the adaptation experiment JNDs were measured under 2 conditions: for an untrustworthy female face (80) following adaptation to an untrustworthy male face (matched for untrustworthiness), and without adaptation. The experimental procedure was identical to that used in experiments one and two.

### General analysis

For each participant and condition in each of the three adaptation experiments, JNDs were computed by first fitting cumulative Gaussians psychometric functions to the data. We divided the resulting standard deviations by $\sqrt{2}$ to give an estimate of the standard deviation on a single interval [because we used a two-interval experimental procedure; (Green and Swets, 1974)]. The resulting values are JNDs because they indicate the % change in untrustworthiness that can be discriminated at the ~76% level.

## Results

### Experiment 1

**Figure 4:**
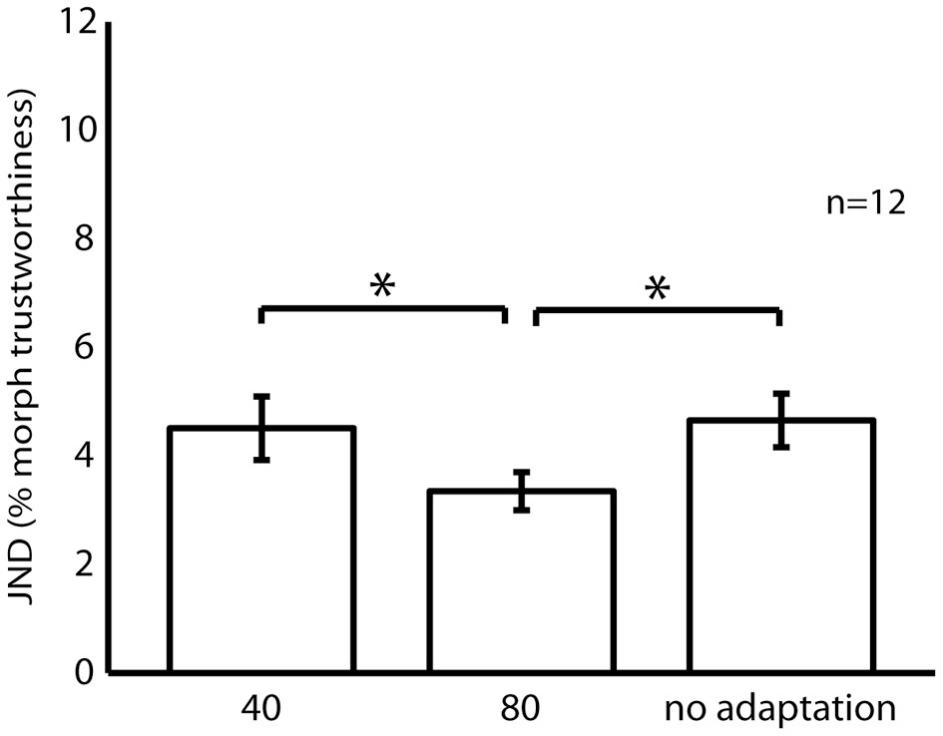
**Trustworthiness discrimination thresholds for an untrustworthy female face (80) following adaptation to a trustworthy female face (40), an untrustworthy female face (80), and no adaptation.** Error bars denote ± SEM. Asterisk denote a significant difference between conditions (^*^p < 0.05).

Experiment 1 measured the effects of adaptation to untrustworthy (80) and trustworthy (40) female faces on discrimination thresholds around an untrustworthy female face (80). Average trustworthiness discrimination thresholds are shown in Figure 4. An ANOVA with adaptation condition as a within subjects factor and participant gender a between subjects factor showed a significant main effect of adaptation condition [*F*_(2, 20)_ = 6.28, *p* < 0.01 η^2^_*p*_ = 0.39]. Planned pair-wise comparisons confirmed that JNDs were smaller when adapting to an untrustworthy face compared to either adapting to a trustworthy face (*p* < 0.05), or no adaptation (*p* < 0.05). The JNDs in the no adaptation and trustworthy adaptation conditions were equivalent (*p* < 0.05). A significant main effect of participant gender [*F*_(1, 10)_ = 15.96, *p* < 0.01 η^2^_*p*_ = 0.62] was observed as female participants had lower discrimination thresholds (*M* = 3.1, *SD* = 1.1) than males (*M* = 5.3, *SD* = 2.0). There was no significant interaction between adaptation condition and participant gender [*F*_(2, 20)_ = 2.52, *p* > 0.05, η^2^_*p*_ = 0.20].

### Experiment 2

**Figure 5:**
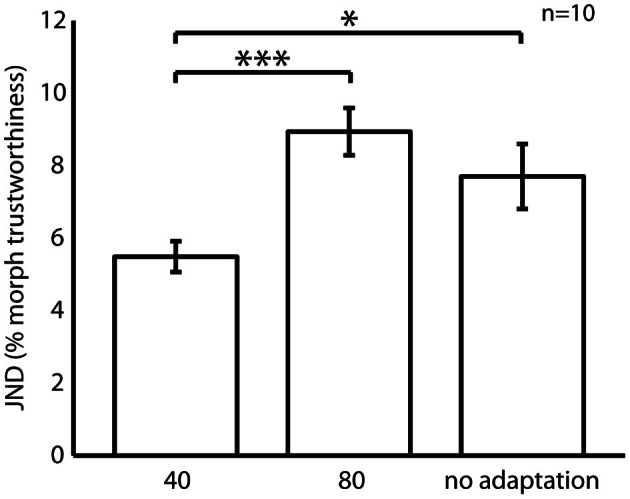
**Trustworthiness discrimination thresholds for a trustworthy female face (40) following adaptation to a trustworthy female face (40), an untrustworthy female face (80), and no adaptation.** Error bars denote ± SEM. Asterisk denote a significant difference between conditions (^*^p < 0.05; ^***^p < 0.001).

Experiment 2 measured the effects of adaptation to untrustworthy (80) and trustworthy (40) female faces on discrimination thresholds for a trustworthy female face (40). Figure 5 shows the trustworthiness discrimination thresholds. As with experiment 1 we analysed discrimination thresholds with ANOVA, and found a significant main effect of adaptation condition [*F*_(2, 16)_ = 11.80, *p* < 0.01 η^2^_*p*_ = 0.60]. Planned pair-wise comparisons confirmed that JNDs were smaller when adapting to a trustworthy face, compared to either adapting to an untrustworthy face (*p* < 0.001), or no adaptation (*p* < 0.05). JNDs did not differ significantly between the untrustworthy and no adaptation conditions (*p* > 0.05). No effect of participant gender [female, *M* = 6.98, *SD* = 2.0; male, *M* = 7.78, *SD* = 4.7; *F*_(1, 8)_ = 0.49, *p* > 0.05 η^2^_*p*_ = 0.06], or interaction between participant gender and adaptation condition [*F*_(2, 16)_ = 0.67, *p* > 0.05 η^2^_*p*_ = 0.08] was observed.

### Experiment 3

Experiment 3 examined the effects of adapting to an untrustworthy male face on discrimination thresholds for an untrustworthy female face (80). For each participant the male adaptor face was matched on untrustworthiness (*M* = 80, *SD* = 5.7) to the female standard face (80). Following adaptation to the untrustworthy male face discrimination thresholds for the female untrustworthy face were significantly lower (*M* = 4.16, *SD* = 1.38) compared to the no adaptation condition [*M* = 4.93, *SD* = 1.81; one-tailed *t*-test, *t*_(14)_ = 1.88, *p* < 0.05]. The reduction in face discrimination thresholds seen when the adapting stimulus gender and identity were different from the test faces (Experiment 3) was 65% of the size of the reduction in discrimination thresholds seen when the adapting stimulus gender and identity were the same as the test faces (Experiment 1). The improvement in face trustworthiness discrimination with adaptation was reduced, but still present when the adapting face was a different identity and gender.

## Discussion

Here we show that adaptation to an untrustworthy or a trustworthy face results in a selective improvement in discrimination thresholds for facial trustworthiness. Adaptation to an untrustworthy face, but not adaptation to a trustworthy face, improves discrimination of untrustworthy faces. Conversely, adaptation to a trustworthy face, but not adaptation to an untrustworthy face, improves the discrimination of trustworthy faces. This selective enhancement of face perception occurs even when the adapting face has a different gender and identity to the subsequent test faces.

Previous studies have indicated that visual adaptation to facial emotion (Engell et al., 2010) and facial trustworthiness (Wincenciak et al., 2013) can bias the perception of facial trustworthiness. We show here, as for low-level stimuli (cf. Clifford and Langley, 1996) that high-level adaptation to facial trustworthiness can have a functional benefit. Exposure to a specific degree of face trustworthiness benefits subsequent perception of similar faces. These improvements in the ability to discriminate the trustworthiness of faces are likely to result from a temporary, but selective, enhancement of the sensitivity of the system underlying the perception of these stimuli.

The improvements in face trustworthiness discrimination are small, but significant and comparable to those found for face gender (Yang et al., 2011) and face orientation adaptation (Chen et al., 2010). The small improvements in sensitivity that we see occurred over a relatively short period (~40 s). As increases in sensitivity are proportional to the length of adaptation, we would expect to see greater improvements in face trustworthiness discrimination over longer periods as might be expected under real world viewing conditions (Clifford and Langley, 1996). Indeed, it has been suggested that prolonged exposure to specific face types may contribute to the “own-race bias,” the ability to better detect differences between individuals of our own race than those of another (Rhodes et al., 2010).

These other previously observed improvements in face discrimination (i.e., identity and gender adaptation) cannot fully explain the effects we observe in this study; although they may have contributed somewhat to the decrease in discrimination thresholds during experiments 1 and 2. However, during experiment 3 participants adapted to a face with a different identity and gender to the test stimuli. Still, we observed a beneficial effect of adaptation to an untrustworthy face on the discrimination of subsequent untrustworthy faces. It is likely, therefore, that a selective enhancement of specific mechanisms underlying the perception of facial untrustworthiness is responsible in part for the effects we observe. These mechanisms thus appear to be independent to both face gender and identity, complementing previous research indicating that (un)trustworthy aftereffects resulting from exposure to one identity face can bias perception of (un)trustworthiness in another identity face (Wincenciak et al., 2013).

It is not entirely clear what mechanism might underlie the greater improvement in (un)untrustworthy face discrimination observed when adapting and test faces have the same gender and identity (Experiments 1 and 2). One possibility is that the perception of face (un)trustworthiness relies on both identity-dependent and identity-independent mechanisms. The results we observed during experiments 1 and 2 might result from the enhanced effect of the simultaneous adaptation of both of these mechanisms. Similarly, Fox and Barton (2007) have shown, using an adaptation paradigm that face expression aftereffects transfer both within and across face identity, arguing for both an identity-dependent and an identity-independent representation of facial expression. Fox and Barton's expression aftereffects are larger when adapting and test face have the same identity, presumably resulting from the adaptation of both identity-dependent and identity-independent representations of facial expression.

An alternate explanation is that the greater improvement in (un)trustworthy face discrimination observed in experiments 1 and 2 results from a simultaneous beneficial influence of gender (Yang et al., 2011) and/or identity (Rhodes et al., 2010) face adaptation. The task of the participants was to explicitly discriminate the degree of untrustworthiness conveyed by the 2 test faces, but we cannot rule out the influence of other factors on this judgment. Facial trustworthiness judgments correlate highly with the emotional valence of faces and can be viewed as an overgeneralization of emotion. Happy people who are more likely to help us and can be approached are viewed as more trustworthy than angry people, who may want to harm us and should be avoided (Oosterhof and Todorov, 2008; Sutherland et al., 2013). Had participants judged which of the test faces was more happy, instead of which was more untrustworthy, we may have found similar results. We have not tested this possibility in the current study because such a finding would not change the interpretation of the results. Adaptation to the perceived valence of the face and other attributes, such as attractiveness that correlate with trustworthiness, are adaptation to trustworthiness, by virtue of the multi-dimensional judgment of this trait.

Female observers were better at discriminating untrustworthy faces, but not trustworthy faces, compared to male observers. This difference in ability might arise as females may pay more attention to these stimuli than males. Previous research has also indicated that there may be a difference in the way that female and male observers process facial (un)trustworthiness. Dzhelyova et al. (2012), during an event related potential (ERP) study of the perception of untrustworthy and trustworthy faces, showed that female observers were more accurate in the perception of facial trustworthiness than male observers. Furthermore, Wincenciak et al. (2013) found that only female observers showed typical repulsive aftereffects, where test stimuli looked *less* like the adapting stimuli, following adaptation to trustworthy and untrustworthy faces. We found no interaction between the gender of the participant and the adapting condition in either experiment 1 or 2. Therefore, the beneficial effect of facial (un)trustworthy adaptation was no different in female and male observers. Such a functional benefit in male observers is interesting given that other research has demonstrated the absence of typical repulsive aftereffects in males (Wincenciak et al., 2013), suggesting adaptation improves, but does not bias, perception of facial (un)trustworthiness in male observers. In future work it would be interesting to examine whether male and female observers show this functional benefit when adapting to and discriminating male facial (un)trustworthiness.

In conclusion, we have shown that adapting to facial (un)trustworthiness can calibrate our visual system, selectively increasing sensitivity, thereby allowing us to detect smaller changes in facial trustworthiness. This process appears to be relatively fast acting, occurring even after exposure to a face for ~1 min. Longer term exposure to faces conveying specific levels of untrustworthiness that might occur with either a specific job (e.g., Police) or from living with particular individuals may confer more pronounced functional and social benefits. Improvements in face discrimination may enhance discrimination between who we should invest in and who we might best avoid (Oosterhof and Todorov, 2008; Sutherland et al., 2013).

### Conflict of interest statement

The authors declare that the research was conducted in the absence of any commercial or financial relationships that could be construed as a potential conflict of interest.
